# Blur Detection Sensitivity Increases in Children Using Orthokeratology

**DOI:** 10.3389/fnins.2021.630844

**Published:** 2021-03-12

**Authors:** Jingjing Xu, Chunwen Tao, Xinjie Mao, Xin Lu, Jinhua Bao, Björn Drobe, Hao Chen

**Affiliations:** ^1^School of Ophthalmology and Optometry, Affiliated Eye Hospital, State Key Laboratory of Ophthalmology, Optometry and Vision Science, Wenzhou Medical University, Wenzhou, China; ^2^WEIRC, Wenzhou Medical University-Essilor International Research Center, Wenzhou, China; ^3^R&D AMERA, Essilor International, Singapore, Singapore

**Keywords:** blur detection sensitivity, orthokeratology, blur adaptation, higher-order aberration, accommodation lag

## Abstract

**Purpose:**

To investigate changes in blur detection sensitivity in children using orthokeratology (Ortho-K) and explore the relationships between blur detection thresholds (BDTs) and aberrations and accommodative function.

**Methods:**

Thirty-two children aged 8–14 years old who underwent Ortho-K treatment participated in and completed this study. Their BDTs, aberrations, and accommodative responses (ARs) were measured before and after a month of Ortho-K treatment. A two forced-choice double-staircase procedure with varying extents of blur in three images (Tumbling Es, Lena, and Street View) was used to measure the BDTs. The participants were required to judge whether the images looked blurry. The BDT of each of the images (BDT_Es, BDT_Lena, and BDT_Street) was the average value of the last three reversals. The accommodative lag was quantified by the difference between the AR and the accommodative demand (AD). Changes in the BDTs, aberrations, and accommodative lags and their relationships were analyzed.

**Results:**

After a month of wearing Ortho-K lenses, the children’s BDT_Es and BDT_Lena values decreased, the aberrations increased significantly (for all, *P* ≤0.050), and the accommodative lag decreased to a certain extent [T(31) = 2.029, *P* = 0.051]. Before Ortho-K treatment, higher-order aberrations (HOAs) were related to BDT_Lena (*r* = 0.463, *P* = 0.008) and the accommodative lag was related to BDT_Es (*r* = −0.356, *P* = −0.046). After one month, no significant correlations were found between the BDTs and aberrations or accommodative lags, as well as between the variations of them (for all, *P* ≥ 0.069).

**Conclusion:**

Ortho-K treatment increased the children’s level of blur detection sensitivity, which may have contributed to their good visual acuity.

## Introduction

Orthokeratology (Ortho-K) has been demonstrated to improve naked visual acuity during the day (Swarbrick, 2006; Khan et al., 2016) by reshaping the corneal surface and to slow myopia progression (Swarbrick, 2006; Le et al., 2010; Santodomingo-Rubido et al., 2015, 2017; Wen et al., 2015; Huang et al., 2016; Chen et al., 2018) by changing the peripheral defocusing state (Queirós et al., 2010; Kang and Swarbrick, 2011; Santodomingo-Rubido et al., 2013). An increasing number of children have been using them in China (Tay et al., 2017), Singapore (Saw et al., 2019), etc., especially for the latter reason.

Although some studies (Queiros et al., 2012; Zhao et al., 2018) have shown that myopic children’s quality of life partially improves with good acceptance of Ortho-K (Wen et al., 2015; Li et al., 2016), visual and optical quality in children wearing Ortho-K lenses deteriorate because of the reshaped cornea and the affected tear film (Li et al., 2018). Previous studies have reported that Ortho-K inevitably increases higher-order aberrations (HOAs) and decreases contrast sensitivity (Hiraoka et al., 2008a,b; Gifford et al., 2013; Vincent et al., 2020). Santolaria et al. (2013) reported that people using Ortho-K tend to complain about distortions from car headlights and bright lights at home. Bad visual and optical quality may cause the perception of blur to some extent. However, it is not common for people receiving Ortho-K to complain about blur in the clinic. Hiraoka et al. (2009b) investigated patient satisfaction with visual outcomes using a visual analog scale. The results showed a relatively high level of patient satisfaction following Ortho-K use, which was associated with pretreatment myopic error and posttreatment uncorrected visual acuity.

Blur is a visual signal that triggers the visual feedback mechanism and plays an important role in the emmetropization process (Norton and Siegwart, 1995). It has been shown that people are likely to adapt to blur if it lasts for a certain period of time (Khan et al., 2013). Therefore, we speculated that children with Ortho-K experience a blur adaptation induced by increased aberrations and greater diurnal variations in vision (Queiros et al., 2012; Guo et al., 2018). Blur adaptation is defined as an improvement in visual resolution after exposure to the blur (i.e., defocusing) without a change in the refractive error (Vera-Diaz et al., 2004; Cufflin et al., 2007). After blur adaptation, contrast sensitivity (Pesudovs and Brennan, 1993), blur sensitivity (Cufflin et al., 2007), and sometimes even the accommodative response (AR) (Vera-Diaz et al., 2004) improve. The blur detection threshold (BDT), that is, the amount of detectable blur equivalent to the depth of focus (Jacobs et al., 1989), is closely related to blur sensitivity.

We present a novel hypothesis that Ortho-K provides good naked visual acuity with good satisfaction, partially because children with Ortho-K undergo a blur adaptation. Since the BDT is a good indicator of blur sensitivity, we evaluate the BDT in children before and after Ortho-K treatment to verify whether the deteriorated visual quality changes their blur perception after wearing Ortho-K lenses. Optical physical parameters, such as accommodative lag and ocular aberrations, might explain the changes in the visual feedback mechanism. The relationship between BDTs and visual quality or accommodative function also needs to be clarified.

To the best of our knowledge, no literature has reported the effect of wearing Ortho-K lenses on blur sensitivity. Therefore, this study aimed to evaluate changes in blur detection sensitivity after Ortho-K treatment by measuring the BDT. Moreover, we probed whether this change is related to aberrations or accommodative lag.

## Materials and Methods

### Participants

This clinical study was conducted at the Eye Hospital of Wenzhou Medical University (Wenzhou, China) between May 2016 and October 2017. Forty-three children aged 8–14 years old wore Ortho-K lenses in both eyes, and the right eyes were initially recruited in this study. The spherical equivalent (SE), calculated by adding half of the cylinder (in minus lens notation) to the sphere, ranging from −1.00 diopters (D) to −6.00 D and no more than −0.75 D of astigmatism. None of the participants had records of ocular, systemic, or neurologic diseases or any type of vision dysfunction. In addition, none of the participants had previously undergone Ortho-K treatment or other myopia control treatments. At enrollment, their visual acuity was correctable to 0.00 (logarithm of the minimum angle of resolution (logMAR) visual acuity) or better, and after a month of Ortho-K treatment, their naked visual acuity during the day should have reached 0.00 (logMAR visual acuity) or better. One child was excluded because of excessive follow-up ARs, and ten children did not have ideal naked visual acuity. In total, thirty-two children completed this study. The participants and their guardians were informed of and understood the adverse reactions, and they all signed the informed consent forms. This study followed the tenets of the Declaration of Helsinki and was approved by the Ethics Committee of Wenzhou Medical University.

### Procedure

All the Ortho-K lenses used in this study were VST-DESIGN with four-zone reverse geometry rigid contact lenses (Lucid, South Korea), included the base curve, reverse curve, alignment curve, and peripheral curve and were composed of Boston XO material, with an oxygen permeability (DK) of 100 × 10^–12^ (cm^2^ × mlO_2_)/(s × ml × mmHg). The lenses had a total diameter of 10.2–10.8 mm, a central zone diameter of 0.6 mm, and a central thickness of 0.23 mm.

**FIGURE 1:**
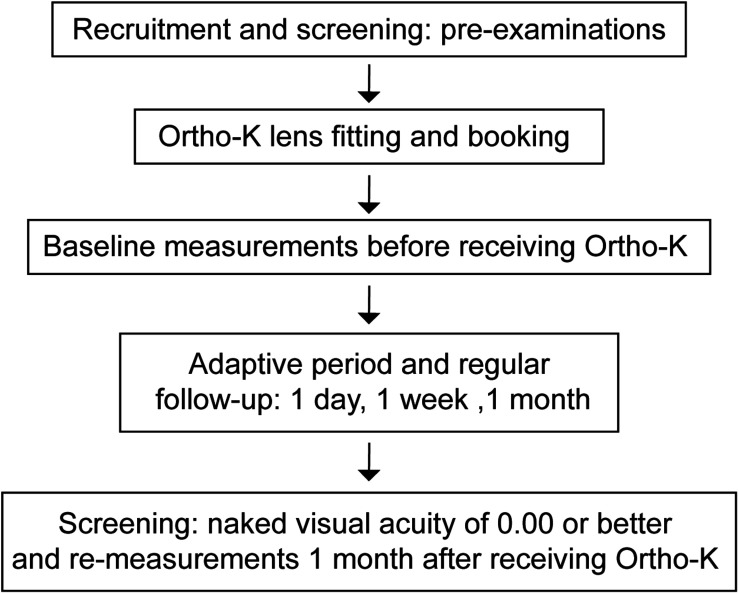
Procedure of this study. Forty-three children who were scheduled to wear Ortho-K lenses had a series of pre-examinations. After lens fitting was performed, the personalized Ortho-K lenses were ordered. The baseline measurements were conducted before they received the Ortho-K lenses. After a month of Ortho-K use, ten children without ideal naked visual acuity were excluded, and the remaining children were measured again. Visual acuity of 0.00 refers to the logMAR visual acuity.

The detailed produce is summarized in Figure 1. The participants first underwent a series of pre-examinations, including subjective refraction, intraocular pressure, axial length, corneal topography and endothelial cell count analysis. Then, lens fitting was performed and evaluated by experienced doctors with slit-lamp biomicroscopy according to the fluorescence pattern and topography. All of the participants were instructed to regularly follow up 1 day, 1 week, 1 month, and then every 3 months after lens delivery.

After enrollment, the BDTs, aberrations, and ARs of the participants were measured before receiving Ortho-K. Measurements were taken on the right eyes, fully corrected with frames, with the left eyes occluded. After a month, the aberrations, ARs and BDTs of the remaining participants were measured for a second time. For most of the Ortho-K children, their corneal parameters as well as refraction (Li et al., 2018; Queirós et al., 2020), visual quality (Stillitano et al., 2008; Santolaria Sanz et al., 2015), and visual performance including naked visual acuity (Lu et al., 2018; Xia et al., 2019) reached a steady state at one month.

#### Blur Detection Threshold (BDT)

The testing procedure was designed to be similar to a computer game, easy for children to complete, which was presented on a 19” LCD Monitor (DELL^TM^ P1914S Monitor, 1280 × 1024 pixels, TX, United States). A neutral gray background provided uniform illumination for the test. The environmental luminance was 155 lux. The heads of the participants were stabilized with a chinrest to keep the eyes one meter away from the screen. This distance was chosen to reduce accommodative lag.

**FIGURE 2:**
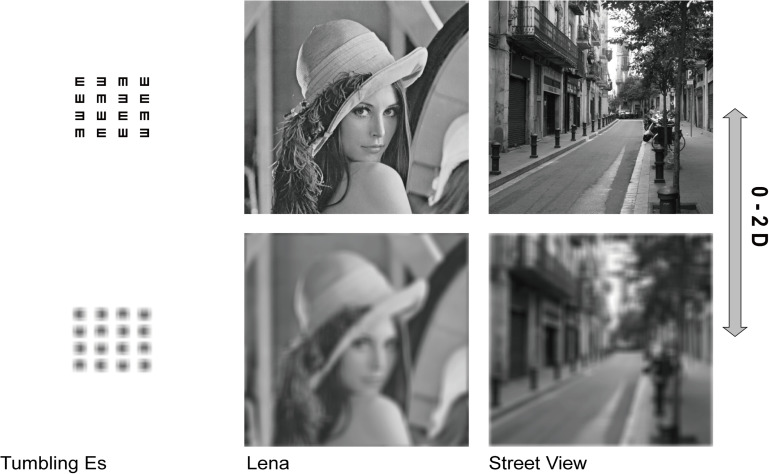
Images used to measure the BDT. Three images with spherical defocus ranging from 0 D ∼ 2 D in steps of 0.01 D are included in the procedure. From left to right: Tumbling Es, Lena, and Street View. The defocus degree of the top images is 0 D and that of the bottom images is 2 D.

The procedure included three images: Tumbling Es, Lena, and Street View (Figure 2), with an average luminance of 50.85, 19.41, and 13.24 cd/m^2^, respectively. Two hundred pictures with spherical defocus ranging from 0 D ∼ 2 D in steps of 0.01 D for each image were obtained by fast Fourier transformation (FFT) based on a pupil radius of 2 mm. The Stiles-Crawford effect (Applegate and Lakshminarayanan, 1993) was considered, with an attenuation factor of *r* = 0.05 mm^2^ and a vertical offset of 0.2 mm.

**FIGURE 3:**
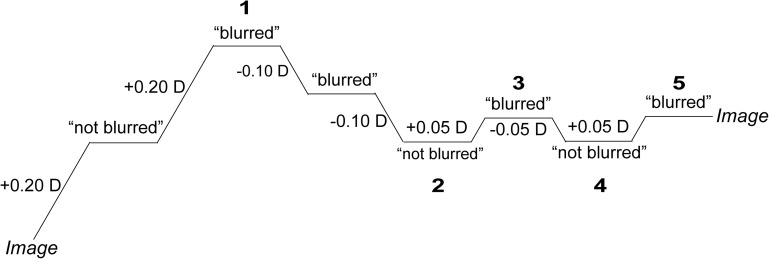
Ascending staircase. The first image in the ascending staircase was the clearest picture with 0 D defocus. The numbers in the figure represent the last five reversals in the procedure, and the BDT is the average value over the last three reversals.

A double-staircase method was used. The two staircases occurred alternately and in parallel, and the three images also appeared in random order. Images with different blur levels were presented for 1 second. The participants were required to judge whether the image looked blurred by clicking on a “blurred” or a “not blurred” button. The first image in the ascending staircase was the clearest one with 0 D defocus, and the first image in the descending staircase was the most blurred with +2 D defocus. The steps of the staircases were 0.20 D until the first inversion, 0.10 D until the second inversion and 0.05 D from the third inversion onward. The BDT was calculated automatically on the last 3 reversals out of 5 (Figure 3).

#### Accommodative Lag

The AR was obtained with a Grand Seiko WAM-5500 open field infrared autorefractor (Grand Seiko, Hiroshima, Japan). One “Tumbling Es” image, a 4 × 4 array of high-contrast letters (the same image used in the BDT procedure with 0 D defocus) was displayed 1 m away from the target. The AR was measured three times in static mode. The mean value was taken as the AR, and the accommodative lag was calculated based on the following formulas for accommodative demand (AD) and the AR (Gwiazda et al., 1993):

$$\begin{array}{l} \mathrm{Accommodative}\,\mathrm{Demand}=\frac{1/\text{DTE}}{1-\mathrm{DLE}*\mathrm{LENS}*2}\\ \mathrm{Accommodative}\,\mathrm{Response}=\mathrm{RI}\\ \mathrm{Accommodative}\,\mathrm{lag}=\mathrm{AR}\,\text{-}\,\mathrm{AD} \end{array}$$

where DTE = the distance from the eye to the target (1 m), DLE = the distance from the lens to the eye (0.12 m), LENS = the signed dioptric power of the lens, and RI = the mean value (calculated above) from the values read from the Grand Seiko device.

These equations correct for the effectivity of a spectacle lens placed 12 mm in front of the eye.

#### Aberrations

The ocular aberrations were measured three times in a dark room with a WASCA wavefront analyzer (Carl Zeiss Meditec, Jena, Germany) through the natural pupil without the use of dilation drugs, and the mean value was used for data analysis. The pupil diameter used for analysis was 5 mm. The total HOAs, trefoil, coma, and spherical aberrations (SA) were used for analysis. The total HOAs were calculated as the root mean square (RMS) of the third- to seventh-order coefficients. The RMS of C_3_^–3^ and C_3_^3^ and the RMS of C_3_^–1^ and C_3_^1^ were calculated to represent trefoil and coma, respectively. The SA was equal to C_4_^0^.

### Data Analysis

SPSS version 22.0 was used for data analysis. The normality of the data was assessed using the Shapiro-Wilk test before conducting parametric tests. Differences between baseline measurements and remeasurements after one month of Ortho-K were tested using paired Student’s t-tests or Wilcoxon signed-rank tests, depending on whether the data were normally distributed. Similarly, correlations between the BDTs and aberrations or accommodative lags, including baseline measurements, remeasurements and their variations, were assessed with Pearson correlation coefficients or Spearman correlation coefficients, depending on whether the data were normally distributed. *P* ≤0.05 was set as the level of significance.

## Results

Thirty-two participants (15 boys and 17 girls), aged 8 to 14 years old (11.05 ± 1.59, mean ± SD), successfully completed the 1-month follow-up examinations. The SE at baseline was >−6.00 and <−1.00 D (−3.57 ± 1.23) and ≥−0.75 and ≤0.00 D (−0.19 ± 0.28) with refractive astigmatism. Before Ortho-K treatment, the AD was 0.92 ± 0.03 D, and the AR was 0.52 ± 0.41 D; 1 month after receiving Ortho-K, the AD was 1.00 ± 0 D, and the AR was 0.79 ± 0.42 D. The mean value of the corrected visual acuity was 0.00 ± 0.02 (logMAR) at baseline, and the naked visual acuity after one month of treatment was 0.04 ± 0.05 (logMAR) (Wilcoxon signed-rank test, Z = −2.982, *P* = 0.003). There were no complaints from the subjects about wearing the Ortho-K lenses.

BDT_Es and BDT_Lena decreased significantly following treatment (*P* ≤0.040). The HOAs, trefoil, coma, and SAs increased significantly (all *P* ≤0.050); accommodative lag decreased from 0.40 ± 0.41 D at baseline to 0.26 ± 0.31 D after 1 month [T(31) = 2.029, *P* = 0.051]. In summary, the results showed that after Ortho-K, the BDTs (BDT_Es and BDT_Lena) and the accommodative lags of the participants decreased, while the aberrations increased (Table 1).

**TABLE 1:** Variations in aberrations, accommodations and BDTs before and one month after Ortho-K treatment.

	*N*	Before (M ± SD)	After (M ± SD)	T/Z	*P*
BDT_Es (D)	32	0.51 ± 0.14	0.47 ± 0.16	2.146	0.040
BDT_Lena (D)	32	0.65 ± 0.18	0.60 ± 0.16	2.321	0.027
HOAs (μm)	32	0.21 ± 0.09	0.54 ± 0.18	–9.501	<0.001
Trefoil (μm)	32	0.07 ± 0.04	0.10 ± 0.06	1.963	0.050
Coma (μm)	32	0.13 ± 0.08	0.30 ± 0.18	–5.028	<0.001
SAs (μm)	32	0.07 ± 0.07	0.35 ± 0.13	4.693	<0.001
Accommodative lag (D)	32	−0.40 ± 0.41	−0.26 ± 0.31	2.029	0.051

*N, number of participants; HOAs, total higher-order aberrations; SAs, spherical aberrations; BDT_ Es, the blur detection threshold for the Tumbling Es image; BDT_Lena, the blur detection threshold for the Lena image; BDT_Street, the blur detection threshold for the Street View image; M ± SD, mean ± standard deviation.*

**FIGURE 4:**
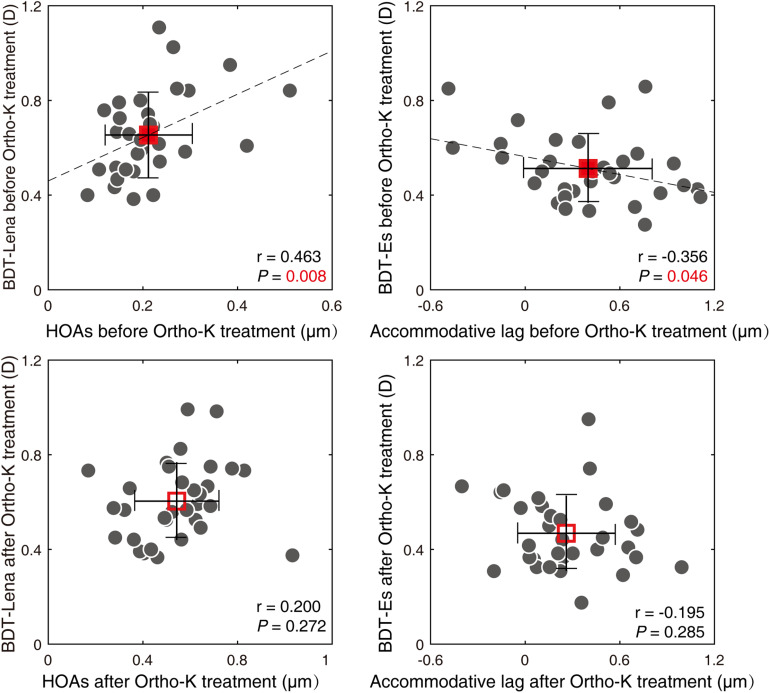
Relationships between BDTs and aberrations and accommodative lag. Two of the BDTs are plotted as a function of aberrations or accommodative lag. Each dot represents the results from one participant. The average of these results is plotted with a square symbol. Red solid square represents pre-treatment, and the hollow one represents post-treatment. The error bars represent the standard deviation. HOAs, total higher-aberrations; BDT_Lena, the blur detection threshold for the Lena image; BDT_Es, the blur detection threshold for Tumbling Es image.

Before Ortho-K treatment, HOAs were significantly related to BDT_Lena (*r* = 0.463, *P* = 0.008), accommodative lag was related to BDT_Es (*r* = −0.356, *P* = −0.046) (Figure 4), and the other possible combinations between aberrations and the BDTs were not significant (all *P* ≥ 0.051). After Ortho-K treatment, there were no relationships among accommodative lag, aberrations and the BDTs (all *P* ≥ 0.069) (Figure 4). The relationships among the variations in accommodative lag, aberrations and BDTs (BDT_Es and BDT_Lena) after Ortho-K treatment were further analyzed, and no significant correlations were found (for all, *P* ≥ 0.201) (Supplementary Figures 1–5).

## Discussion

This is the first study that investigated the effect of Ortho-K on the BDTs and analyzed the correlations among BDTs, aberrations and accommodative lags. To prevent the influence of nonideal visual acuity on BDT evaluation, participants whose naked visual acuity was 0.00 logMAR or better at the one-month follow-up examination were included, and the experiments were all conducted in the morning. These parameters were evaluated before and one month after Ortho-K treatment because the naked visual acuity of the subjects may become worse than 0.00 (LogMAR visual acuity) at a long-term follow-up visit due to myopia progression (Wen et al., 2015; Na and Yoo, 2018). The results showed that the blur detection sensitivity for Es and Lena in the children improved after Ortho-K treatment. Previous studies (Battaglia et al., 2004; Wang et al., 2006; Venkataraman et al., 2015) have indicated that the blur adaptation effect exists only in fovea, therefore more arresting images are a better target for BDT measurement. According to our previous study (Lu et al., 2016), the Es and Lena images are more suitable for measuring the blur adaptation effect.

In this study, aberrations (HOAs, trefoil, coma and SAs) increased significantly, demonstrating that the visual quality in the children worsened after Ortho-K treatment. The HOAs increased from 0.21 ± 0.09 μm to 0.54 ± 0.18 μm, coma increased from 0.13 ± 0.08 μm to 0.30 ± 0.18 μm, and the SAs increased from 0.07 ± 0.07 μm to 0.35 ± 0.13 μm after one month. Our results were consistent with those from previous articles. Lian et al. (2014) found that 30 days after wearing Ortho-K lenses, HOAs increased from 0.27 ± 0.12 μm to 0.69 ± 0.21 μm, coma increased from 0.14 ± 0.10 μm to 0.36 ± 0.26 μm, and SAs increased from 0.06 ± 0.11 μm to 0.44 ± 0.20 μm. The reason for the minor difference is likely the different pupil diameters used for analysis; we used 5 mm diameters, while their study used 6 mm diameters. Analysis with a larger pupil diameter can result in larger aberrations (Wang et al., 2003); therefore, the values in this study were slightly smaller than those of the other study. Indeed, the changes observed in our study and in the study by Lian et al. are similar. Gifford et al. (2013) also reported that ocular HOAs, SAs and coma increased considerately after 7 nights of wearing Ortho-K lenses. Studies have shown that increased HOAs in Ortho-K Children can be induced by changes in the corneal surface and treatment zone decentration (Berntsen et al., 2006; Hiraoka et al., 2009a; Gifford et al., 2013).

Few studies have investigated the blur sensitivity of Ortho-K lens wearers, especially in children. Because of the high correlation between the BDTs and HOAs before Ortho-K treatment, there is reason to believe that the blur detection ability is affected by visual inputs not only from lower-order aberrations such as defocus and astigmatism but also from HOAs. However, after Ortho-K treatment, the HOAs significantly increased, while the BDTs did not increase but significantly decreased. This phenomenon seems to be an unintended variation in which BDTs changed in the opposite direction as the HOAs after Ortho-K, whereas they were positively correlated before Ortho-K. The correlation between the BDTs and HOAs did not exist any more after Ortho-K treatment because HOAs were variously affected by corneal shape, lens parameters, lens location et al. The changes of BDTs induced by HOAs were also individually different. We suggest that the variations in BDTs are due to blur adaptation, which could be induced by blur perception. Labhishetty et al. (2019) found there were no significant differences in BDTs between progressive myopic children and their non-myopic peers, which could be explained by compensation in higher visual processes for poor retinal image quality. Hence, the amount of BDT variations induced by additional blur caused by Ortho-K treatment was small, however, the difference is significant because the measurements are quite repeatable and precise. In present study, BDT_Es decreased by 0.04 D and BDT_Lena decreased by 0.05 D. The value is even higher than that with the blur adaptation effect induced with a plus lens. In our previous study, after blur adaptation with 2-diopter plus lens, BDT_Es decreased by 0.023 D and BDT_Lena decreased by 0.035 D (Lu et al., 2016)). Therefore the same targets were used in this study to evaluate the BDTs.

The markedly increased HOAs (0.21 vs. 0.54 μm) indicate deteriorated visual quality, which could lead to the subjects’ blur perception. This unnatural blur may induce blur adaptation in children. The decreased BDTs could be explained as a blur adaptation effect. Several studies have indicated that after blur adaptation, blur sensitivity is significantly improved (Cufflin et al., 2007; Lu et al., 2016). The phenomenon wherein the BDT decreases after blur adaptation was also demonstrated in our previous study (Lu et al., 2016). Mon-Williams et al. indicated that “neural compensation” may be achieved in children after receiving Ortho-K (Mon-Williams et al., 1998). Therefore, the ability to discriminate blur signals in children must have been improved after wearing Ortho-K lenses for 1 month.

Another piece of evidence indicating blur adaptation was the decrease observed in the accommodative lag after Ortho-K treatment in our study. The decrease in the accommodative lag was essentially consistent with but less than the change observed in Han et al. (2018)’study as a result of a smaller AD. Blur is the main signal that drives accommodation. Previous studies have suggested that accommodation is related to blur adaptation. One such study (Vera-Diaz et al., 2004) reported that individuals with myopia showed a significant increase in the near AR (33 cm) after a 3-minute blur adaptation exercise. Le et al. (2010) indicated that blur adaptation in both myopic and emmetropic participants would elevate instability of the AR. The increase in accommodative lag in individuals with myopia or premyopia might be due to the decrease in blur stimuli during near work (Goss and Wickham, 1995), indicating that blur should be an effective signal for increasing the AR. We speculate that the decreased accommodative lag is induced by blur adaptation, which is caused by the deterioration in visual quality following Ortho-K in children. The presence of similar BDTs in myopic and emmetropic children but a larger accommodative lag in myopic eyes means the low sensitivity to defocus could be compensated by some form of an adjustment in the higher visual processes (i.e., blur adaptation) to preserve or increase the subjective perception (Labhishetty et al., 2019).

In another way, it was indicated (Wang et al., 2018) that the increased coma was highly related to the decentration of orthokeratology lenses. Oshika et al. (2002) found that coma-like aberration and corneal multifocality had significant positive correlations with the magnitude of apparent accommodation in pseudophakic eyes. His group (Hiraoka et al., 2015) reported later that the ocular coma-like aberration and corneal multifocality significantly increased in children undergoing orthokeratology and the change in coma-like aberration was highly associated with the change in corneal multifocality. In this study, the increased coma-like aberration and increased range of focus after orthokeratology treatment also might induce greater accommodative response, that is less accommodative lag, although no correlation was found between HOAs and accommodative lag as well as variations of them (Supplementary Figure 6).

Based on the close correlation between the BDTs and HOAs as well as the accommodative lag before Ortho-K treatment and the variable trend after treatment, we speculate that the significantly worsened visual quality could result in blur, leading to a blur adaptation effect and therefore a decrease in the BDTs and accommodative lags. A smaller BDT, which represents higher blur sensitivity, may promote the ability to recognize visual targets, which could explain the subjective sharpness and visual acuity of the children wearing Ortho-K lenses despite their poor visual quality, provided that a large number of lower-order aberrations were excluded. A number of studies have affirmed that blur could induce the emmetropization process and be closely related to myopic progression (Norton and Siegwart, 1995; Read et al., 2010; Chakraborty et al., 2012; Wang et al., 2016; Delshad et al., 2020). We have verified that blur adaptations do exist in children wearing Ortho-K lenses, and the role of blur adaptation in the myopia control effect of Ortho-K needs further study.

In conclusion, this study confirmed that after receiving Ortho-K lenses, aberrations increased, blur sensitivity increased, and the accommodative lag decreased. The correlation among these visual function parameters supports the notion that increased blur sensitivity is related to deteriorated visual quality caused by cornea reshaping by Ortho-K. Children with Ortho-K undergo blur adaptation, contributing to their good visual acuity in the daytime.

## Data Availability Statement

The raw data supporting the conclusions of this article will be made available by the authors, without undue reservation.

## Ethics Statement

The studies involving human participants were reviewed and approved by Ethics Committee of Wenzhou Medical University. Written informed consent to participate in this study was provided by the participants’ legal guardian/next of kin.

## Author Contributions

HC and JX conceived the experiments. JX and BD determined the experimental methods. CT, XM, and XL performed the experiments. CT and JX analyzed the data and interpreted the data. CT, JX, and JB wrote the manuscript. BD and HC modified the manuscript. All authors contributed to manuscript revision, read and approved the submitted version.

## Conflict of Interest

BD was employed by the company Essilor International in Singapore. The remaining authors declare that the research was conducted in the absence of any commercial or financial relationships that could be construed as a potential conflict of interest.
