# Supplementary material for: Blur Detection Sensitivity Increases in Children Using Orthokeratology
Source: Front Neurosci. 2021 Mar 12;15:630844. doi: 10.3389/fnins.2021.630844 (PMC8006440; doi:10.3389/fnins.2021.630844)
Supplement: Supplementary file 1 [file Data_Sheet_1.pdf]

Supplementary file for

Blur detection sensitivity increases in children using orthokeratology.

Jingjing Xu[a,b#], Chunwen Tao[a#], Xinjie Mao[a,b], Xin Lu[a], Jinhua Bao[a,b], Björn Drobe[b,c], Hao Chen[a,b]

[a].School of Ophthalmology and Optometry, Affiliated Eye Hospital, State Key Laboratory of Ophthalmology, Optometry and Vision Science, Wenzhou Medical University, Wenzhou, Zhejiang, People's Republic of China

[b].WEIRC, Wenzhou Medical University-Essilor International Research Center, Wenzhou, Zhejiang, People's Republic of China

[c].R&D AMERA, Essilor Internal, Singapore, Singapore

# contributed equally

* Corresponding author: chenhao@mail.eye.ac.cn

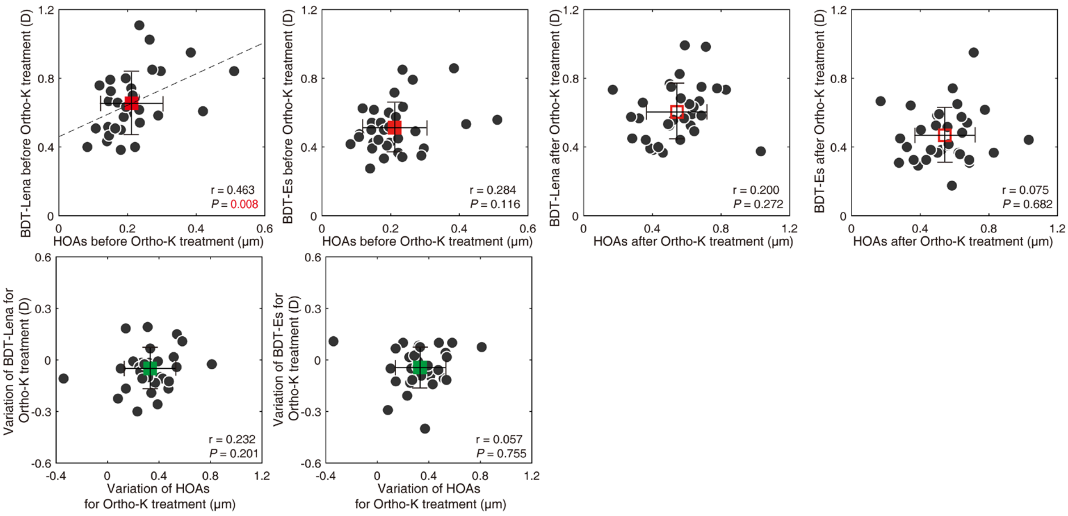

Figure 1. Relationships between HOAs and the BDTs

Two of the BDTs are plotted as a function of HOAs. Each dot represents the results from one participant. The average of these results is plotted with a square symbol. Red solid square represents pre-treatment, red hollow square represents post-treatment and the green one represents the variations. The error bars represent the standard deviation. HOAs, total higher-aberrations; BDT_Lena, the blur detection threshold for the Lena image; BDT_Es, the blur detection threshold for Tumbling Es image.

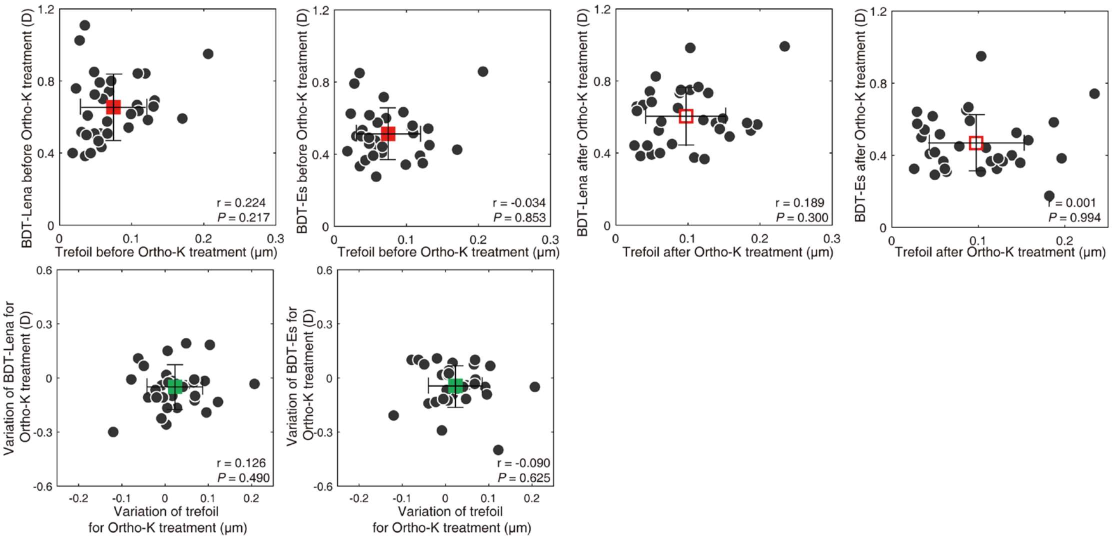

Figure 2. Relationships between trefoil and the BDTs

Two of the BDTs are plotted as a function of trefoil. Each dot represents the results from one participant. The average of these results is plotted with a square symbol. Red solid square represents pre-treatment, red hollow square represents post-treatment and the green one represents the variations. The error bars represent the standard deviation. BDT_Lena, the blur

detection threshold for the Lena image; BDT_Es, the blur detection threshold for Tumbling Es image.

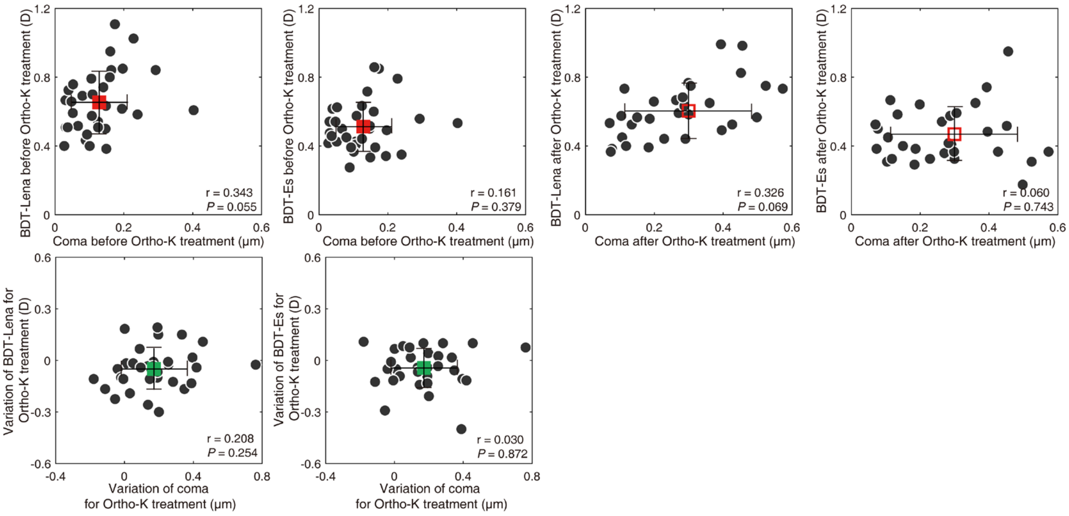

Figure 3. Relationships between coma and the BDTs

Two of the BDTs are plotted as a function of coma. Each dot represents the results from one participant. The average of these results is plotted with a square symbol. Red solid square represents pre-treatment, red hollow square represents post-treatment and the green one represents the variations. The error bars represent the standard deviation. BDT_Lena, the blur detection threshold for the Lena image; BDT_Es, the blur detection threshold for Tumbling Es image

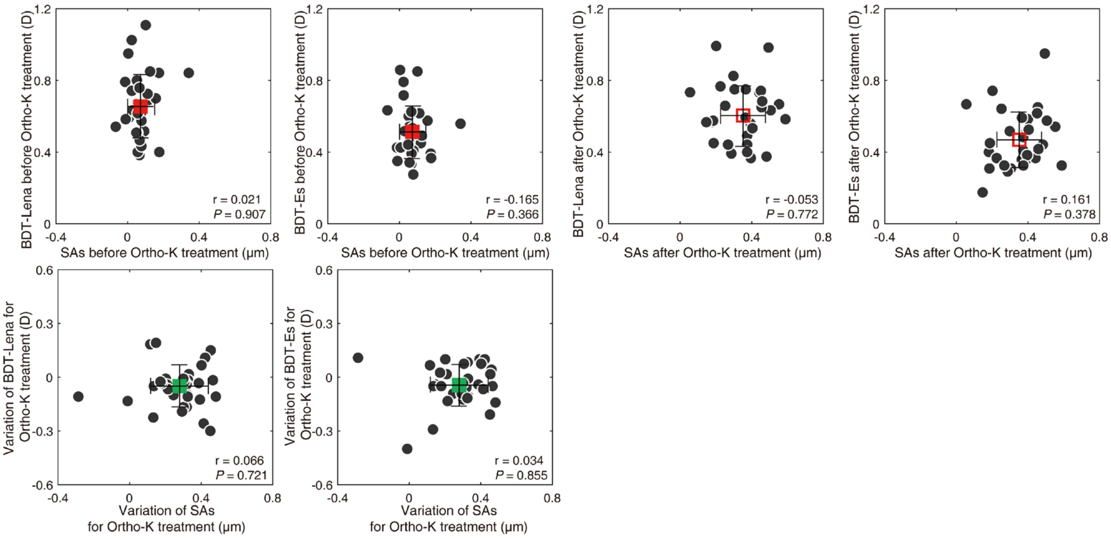

Figure 4. Relationships between SAs and the BDTs

Two of the BDTs are plotted as a function of trefoil. Each dot represents the results from one participant. The average of these results is plotted with a square symbol. Red solid square represents pre-treatment, red hollow square represents post-treatment and the green one

represents the variations. The error bars represent the standard deviation. SA, spherical aberration; BDT_Lena, the blur detection threshold for the Lena image; BDT_Es, the blur detection threshold for Tumbling Es image.

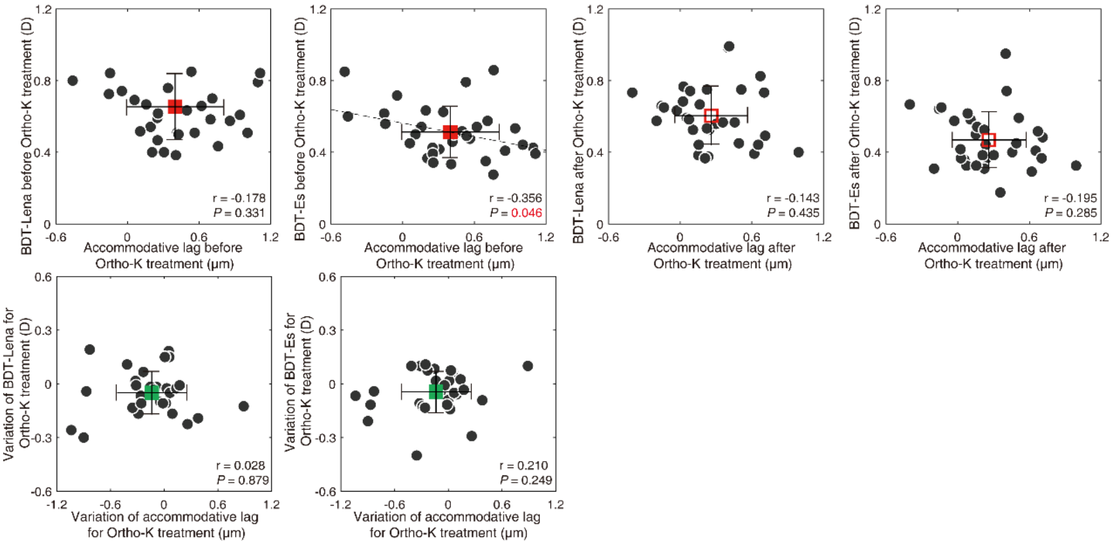

Figure 5. Relationships between accommodative lag and the BDTs

Two of the BDTs are plotted as a function of trefoil. Each dot represents the results from one participant. The average of these results is plotted with a square symbol. Red solid square represents pre-treatment, red hollow square represents post-treatment and the green one represents the variations. The error bars represent the standard deviation. BDT_Lena, the blur detection threshold for the Lena image; BDT_Es, the blur detection threshold for Tumbling Es image.

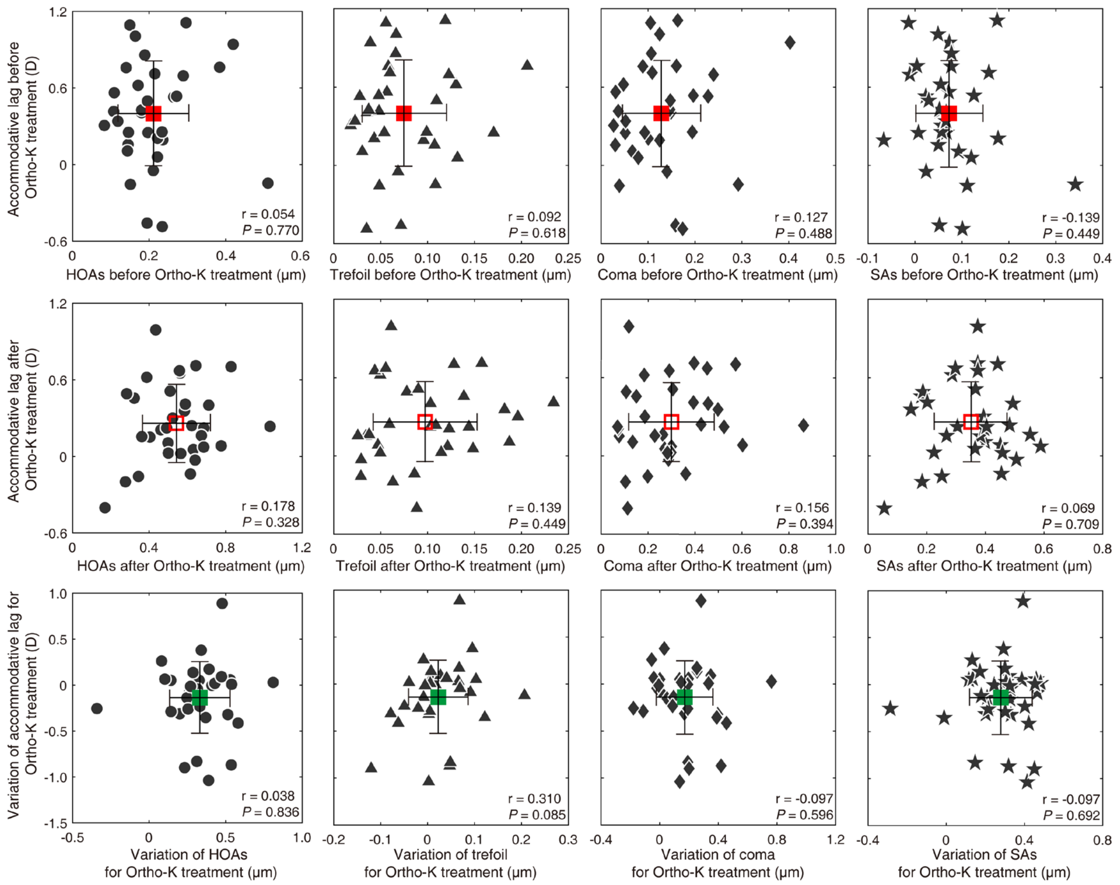

Figure 6. Relationships between accommodative lag and the HOAs, trefoil, coma, SAs. Accommodative lags are plotted as a function of HOAS, trefoil, coma and SAs. Each dot represents the results from one participant. Circle, triangle, diamond and pentagram represent HOAs, trefoil, coma and SAs respectively. The average of these results is plotted with a square symbol. Red solid square represents pre-treatment, red hollow square represents post-treatment and the green one represents the variations. The error bars represent the standard deviation. BDT_Lena, the blur detection threshold for the Lena image; BDT_Es, the blur detection threshold for Tumbling Es image.
